# Autophagy: Friend or Foe in Breast Cancer Development, Progression, and Treatment

**DOI:** 10.4061/2011/595092

**Published:** 2011-09-08

**Authors:** Damian E. Berardi, Paola B. Campodónico, Maria Ines Díaz Bessone, Alejandro J. Urtreger, Laura B. Todaro

**Affiliations:** Research Area, Institute of Oncology “Angel H. Roffo”, University of Buenos Aires, C1417DTB Buenos Aires, Argentina

## Abstract

Autophagy is a catabolic process responsible for the degradation and recycling of long-lived proteins and organelles by lysosomes. This degradative pathway sustains cell survival during nutrient deprivation, but in some circumstances, autophagy leads to cell death. Thereby, autophagy can serve as tumor suppressor, as the reduction in autophagic capacity causes malignant transformation and spontaneous tumors. On the other hand, this process also functions as a protective cell-survival mechanism against environmental stress causing resistance to antineoplastic therapies. Although autophagy inhibition, combined with anticancer agents, could be therapeutically beneficial in some cases, autophagy induction by itself could lead to cell death in some apoptosis-resistant cancers, indicating that autophagy induction may also be used as a therapy. This paper summarizes the most important findings described in the literature about autophagy and also discusses the importance of this process in clinical settings.

## 1. Autophagy: Basic Concepts

Autophagy is the primary intracellular catabolic process responsible for long-lived proteins and organelles degradation and recycling, whereas the ubiquitin/proteosome system is the major cellular pathway responsible for short-lived proteins degradation [1]. Autophagy is an evolutionarily conserved mechanism throughout macromolecules, ribosomes, and organelles are degraded.

**Figure 1:**
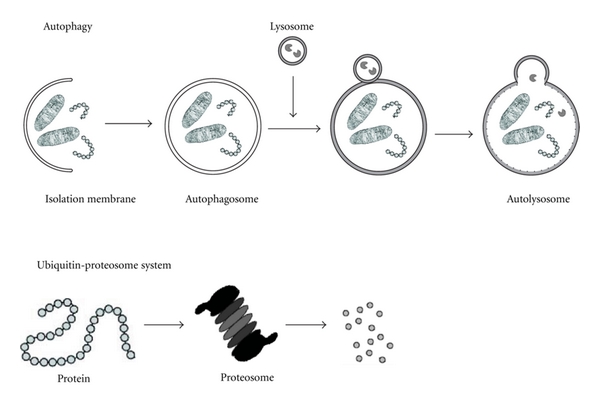
Different mechanisms to recycle molecules and organelles in the eukaryotic cell. Eukaryotes have two major protein degradation systems within cells. One is the ubiquitin-proteasome system, which accounts for the selective degradation of most short-lived proteins. The other is the autophagy, the primary means for the degradation of cytoplasmic constituents in the lysosome.

Initial steps include vesicle nucleation (isolation of the membrane), vesicle elongation, and completion of the double-membrane vesicle [2]. In autophagy, the cytosolic elements that must be degraded are sequestrated by an isolating double-membrane vesicle of nonlysosomal origin that is sealed, creating an autophagic vacuole or autophagosome. Fusion of lysosomes with autophagosomes provides the enzymes required for degradation of sequestrated components [3]. The initial phagophores are formed from the endoplasmic reticulum, and they act surrounding and packing organelles to form autophagosomes [4] (Figure 1).

Recently, autophagy emerged as a multifunctional pathway activated in response to microenvironmental stress, intracellular damage caused by hypoxia, chemotherapeutic agents, virus infections, and toxins. Autophagy may also have a role in cell death, as cancer cells often develop mutations that confer resistance to apoptosis. Nonapoptotic forms of programmed cell death (PCD) might be targeted for novel approaches [5, 6].

## 2. Physiological Functions of Autophagy Process

Autophagy is considered a physiological mechanism that may serve for temporary cell survival and is triggered by starvation, such as amino acid and nutrient deprivation, hypoxia, and metabolic stress [3].

Recent studies have demonstrated the existence of a nonapoptotic form of programmed death called autophagic cell death, which is now considered as programmed cell death (PCD II). Although autophagy was initially described as a protective mechanism allowing cell survival and generating nutrients and energy, other studies have demonstrated that continuous stress can also promote PCD II [2].

### 2.1. Role of Autophagy in Normal Mammary Gland Development

Several works have proved that autophagy is implicated in normal mammary gland development.

In mammalian, the mammary gland expresses its maximum growth potential maturity after pregnancy and during lactation. The cycle of proliferation-differentiation-regression is repeated at each gestation and can be reproduced in culture systems *in vitro.* A deeper understanding of how growth and differentiation of the mammary tissue are regulated can complement the knowledge of the developmental process as well as the treatment and prevention of mammary cancers [7].

PCD is an essential physiological process operating at all stages of mammary gland remodeling. During mammary gland involution, the extracellular matrix (ECM) and alveolar basement membrane are degraded. Also, the alveoli lose their structural integrity, and massive death of mammary epithelial cells is observed. PCD I (apoptosis) is responsible for cell loss during mammary gland involution [8, 9]. However, there is a lot of evidence suggesting that not only PCD I, but also PCD II is observed in mammary epithelial cells.

*In vitro* and *in vivo* studies of bovine mammary gland physiology have revealed that an enhanced process of autophagy is observed at the end of lactation and during dry periods [10, 11]. It is manifested by the increased expression of Beclin 1 and the higher number of cells with typical morphological features of autophagy. Furthermore, 3D model of bovine mammary epithelial cells grown on Matrigel showed that during the development and differentiation of mammary acini, the level of membrane-bound microtubule-associated protein chain 3 (LC3) was increased [12, 13]. This protein is a well-known autophagy marker.

## 3. Autophagy Marker

The development of targeted small molecule inhibitors, like those used for PI3K-AKT-mTOR pathway, has presented a molecular link between the disruption of this signaling cascade and the autophagy process. The cellular consequence of stimulating or inhibiting autophagy in cancer cells is not completely understood, so it is important that this process be monitored, along with antiproliferative and apoptotic biomarkers, in the preclinical setting. 

LC3 is considered as a specific autophagy marker [14]. After the synthesis of LC3, this molecule is cleaved to form LC3-I, and upon induction of autophagy, LC3-I is conjugated to the lipid phosphatidylethanolamine to form LC3-II, which is tightly bound to the membrane of the autophagosome [15]. Immunoblotting assessment of LC3 expression is an easy method to predict autophagic activity of mammalian cells, because the amount of LC3-II correlates with the number of autophagosomes [13, 16–18]. The product of autophagic conversion of LC3, LC3-II, tightly associates with the autophagosome membrane and migrates faster than LC3-I on SDS-PAGE. Therefore, LC3 immunoblotting may detect two bands: LC3-I with an apparent mobility of 18 kDa and LC3-II with an apparent mobility of 16 kDa.

## 4. Controversial Role of Autophagy in Malignant Transformation

Autophagy could be associated with various pathological conditions including, cardiomyopathy, muscular diseases, neurodegenerative disorders, and cancer.

### 4.1. Autophagy as a Tumor-Suppressor Mechanism

Studies in different cells lines have shown that cancer cells express lower levels of the autophagy-related proteins LC3-II and Beclin 1 than normal epithelial cells [19, 20]. Besides, while heterozygous disruption of BECN1 gene promotes tumor development [19], the overexpression inhibits tumorigenesis [21], supporting the idea that defective autophagy or autophagy inhibition plays a role in malignant transformation. BECN1 gene is deleted in about 50% of breast cancers [21, 22]. In addition, reduced expression of Beclin1 has been reported in other types of cancers such as colon and brain tumors [23, 24]. Overall, the data suggest that a defective autophagic process is clearly linked to cancer development.

The most important evidence linking dysfunctional autophagy and cancer comes from studies demonstrating that autophagy inhibition in mice, by disruption of BECN1, increases cellular proliferation as well as mammary hyperplasia and accelerates tumor development. In addition, transfection of MCF-7 breast cancer cells, that express low levels of Beclin 1, with BECN1 gene, inhibits growth and tumor formation [21]. These results suggest that Beclin 1 is a haploinsufficient tumor suppressor and defective autophagy may be critical for cells malignant transformation [19].

In contrast to apoptosis, PCD II, in general, is caspase independent, does not involve classic DNA laddering, and is believed to be a result of an extensive autophagic degradation of intracellular content [25]. Studies also suggest that apoptosis and autophagy are linked by effectors proteins (e.g., Bcl-2, Bcl-XL, Mcl-1, ATG5, and p53) and common pathways (e.g., PI3K/Akt/mTOR, NF*κ*B, and ERK) [5, 26, 27]. For example, p53 activation triggers starvation response in primary mouse embryonic fibroblasts, which is marked by activation of AMPK (AMP-activated kinase) that inhibits mTOR pathway. In other tissues and cells, p53 may communicate with mTOR pathway by the upregulation of the PTEN and TSC2 genes [28].

There is evidence that autophagy may function as a PCD II in cancer cells in which apoptosis is defective or hard to induce [29]. Therefore, it is reasonable to propose that the induction of autophagic cell death may be used as a therapeutic strategy for cancer treatment.

### 4.2. Autophagy as a Survival and Drugs-Resistance Mechanism

The physiological function of autophagy is related to the maintenance of cellular homeostasis under cellular stress. Utilizing autophagy as a survival mechanism in the severe tumor microenvironment, which is highly hypoxic and acidic, may favor the development of cancer cells.

It was observed that a high number of antineoplastic therapies, radiation therapy, chemotherapy (e.g., doxorubicin, temozolomide, and etoposide), histone deacetylase inhibitors, arsenic trioxide, TNF*α*, IFN*γ*, imatinib, rapamycin, and antiestrogen hormonal therapy (e.g., tamoxifen) induce autophagy, and this induction act as a protective and prosurvival mechanism in human cancer cell lines [20]. In fact, the therapeutic efficacy of these agents can be increased if autophagy is inhibited [30–35]. Other studies have shown that a tumor necrosis factor (TNF) family ligand-tumor necrosis factor-related apoptosis-inducing ligand (TRAIL), induces autophagy in epithelial cells and that TRAIL inhibition promotes luminal filling, when it is combined with Bcl-xL-mediated inhibition of apoptosis [36].

Altogether, disruption of autophagy is involved in diverse human diseases including cancer. In particular, the regulation of autophagy in cancer cells is complex, since it can enhance tumor cell survival in response to certain stresses, but it can also act to suppress the initiation of tumor growth. In contrast to its protective role, inhibition of autophagy through specific gene inactivation can promote tumorigenesis [2].

## 5. Autophagy as a Therapeutic Target in Cancer Patients

Autophagy, which could be either cytoprotective or cytotoxic, is often observed in tumor cells in response to chemotherapy [49] (Table 1).

Mutations or allelic loss of Beclin 1 is frequently found in breast, ovarian, and prostate cancer [9, 10]. Beclin 1 provided the first connection between cancer and autophagy [2]. It has been suggested that autophagy plays an important role in chemoresistance of cancer to some therapeutic agents that typically induce an apoptotic response [39].

### 5.1. Role of Autophagy in Resistance to Therapy

#### 5.1.1. Autophagy in Breast Cancer Treatment: Endocrine Therapy and Endocrine Resistance

Endocrine therapy is administered as an antiestrogen (AE) like Tamoxifen (TAM) or Fulvestrant (FAS; Faslodex; ICI 182,780) or as aromatase inhibitor (AI) such as Letrozole or Exemestane. It is less toxic and potentially more effective therapy in management of hormone-dependent breast cancers. Antiestrogens, and TAM in particular, have been the “gold standard” first-line endocrine therapy for over 30 years [50]. It is likely that the clinical experience with this drug exceeds 15 million patient years [51]. Moreover, TAM is the only single agent with demonstrated efficacy in both premenopausal and postmenopausal women with invasive breast cancer. Unfortunately, until nowadays, the inability of endocrine therapies to cure many women with ER+ disease remains.

The precise mechanism by which breast cancer cells die following estrogen withdrawal (or AI treatment) or AE treatment is unclear. For example breast cancer cells respond to AEs and to estrogen withdrawal even if they have a mutated p53 [52, 53]. Although cell death is one of the apoptosis endpoints, there are earlier events initiated by autophagy signals that could be explaining these treatment responses [53].

Autophagy has been implicated by the induction of this mechanism in response to endocrine therapy. Recent studies showed that endocrine therapy modifies the number of autophagosomes, increases LC3 protein cleavage, and reduces expression of p62 [54]. Consistent with other reports, PCD II is associated with the growth inhibitory effects of endocrine therapy in breast cancer cells [32, 55, 56].

It remains unclear whether autophagy or apoptosis dominates as the cell-death mechanism or whether this varies among different breast cancer cells.

While there is currently no definitive understanding of the primary cell-death mechanisms either in experimental models or breast tumors in women about the relative importance of endocrine therapy-induced changes in proliferation, there are potentially important implications for the underlying biology of the cancer cells. If the primary driver of response as seen in tumor shrinkage is a reduction in proliferation, this will leave many cells alive and still metabolically active. Surviving cells have the ability to adapt to the endocrine-induced stress and eventually overcome the proliferative blockade and grow so that, they will become resistant [57–59]. It is quite possible that autophagy allows breast cancer cells to adapt to endocrine-induced stress and survive. Evidence showed that inhibition of autophagy sensitizes breast cancer cells to TAM [32].

#### 5.1.2. The Role of Autophagy in Bortezomib Treatment against Breast Cancer

The 26S proteasomes are multicatalytic protease complexes consisting of a 20S catalytic core and a regulator 19S subunit responsible for most nonlysosomal intracellular degradation [60]. The dipeptide boronic acid Bortezomib is a selective and potent inhibitor of the 26S proteasome that reversibly inhibits the proteasomal chymotrypsin-like activity [60, 61].

The inhibition of the 26S proteasome by Bortezomib may lead to the accumulation and aggregation of misfolded proteins in the endoplasmic reticulum lumen resulting in the activation of an unfolded protein response (UPR) through the action of three key endoplasmic reticulum-resident transmembrane proteins, pERK, IRE1, and ATF6 [62–64]. The activated protein pERK is a member of a family of protein kinases that phosphorylates the subunit of the cytosolic eukaryotic translation initiation factor eIF2a, resulting in a reduced global protein synthesis and in a preferential translation of selected mRNAs including activating transcription factor 4 (ATF4) [63, 64]. Some reports have identified endoplasmic reticulum stress and the eIF2a/pERK pathway as potent inducers of macroautophagy where it promotes cell survival [65–67].

A recent study in MCF7 cell line showed that during Bortezomib treatment, LC3B protein and mRNA levels increased significantly in a dose and time-dependent manner. The increase of autophagy in Bortezomib-treated cells was dependent on upregulation of LC3B by ATF4 [68]. In addition, MCF7 cells transfected with RNAi specific to LC3B, ATF4, or pERK were more sensitive to Bortezomib treatment. Furthermore, the loss of LC3B or ATF4 was associated with a significant increase in dead cells staining for both Annexin V and propidium iodide after 48 and 72 hours of treatment [68].

From a clinical point of view, it would be an attractive possibility to target autophagy to enhance the response of breast cancer to Bortezomib and sensitize to environmental stress that normally occurs in solid tumors. 

However, clinical experience with Bortezomib has shown limited activity against breast cancer when used as a single agent [61].

#### 5.1.3. The Role of Autophagy in Trastuzumab Treatment against Breast Cancer

Trastuzumab (Tzb and Herceptin) was the first immunotherapeutic drug for the treatment of breast carcinomas overexpressing the HER2 (erbB-2) oncogene that was successful [69–73]; however, the mechanisms that could explain de novo and acquired resistance to anti-HER2 monoclonal are not well understood. Proposed mechanisms for innate or acquired resistance to Tzb include steric inhibition of Tzb binding to the extracellular domain (ECD) of the HER2 tyrosine kinase receptor imposed by other extracellular factors such as the glycoprotein mucin 4 (MUC-4) [74, 75].

Recent work showed that Tzb-resistant HER2-positive breast cancer cells (SKBR3 cell line) exhibit increased basal autophagy through an increase in LC3-II expression compared to Tzb-naïve SKBR3 parental cells, suggesting that acquired Tzb autoresistance of Tzb-conditioned cells is accompanied by increased autophagy. Furthermore, inhibition of formation of preautophagosomal structure upon treatment with 3-methyladenime (3-MA), a pharmacological inhibitor of autophagy, notably reduced cell viability in Tzb-resistant HER2-positive breast cancer cells but not in Tzb-naïve SKBR3 parental cells [76]. To provide additional evidence that autophagy plays a critical survival role in enabling Tzb-insensitive high-rates of cell proliferation in Tzb-refractory cells, the potent and highly sequence-specific mechanism of RNA interference (RNAi) was used to block LC3-dependent autophagosome formation. This assay avoided any off-target side effects that may confound interpretation of the results obtained with autophagy inhibitors, showed that TzbR cells were extremely fragile [76]. These findings, altogether, clearly established that hyperactivation of basal autophagy plays an essential survival role in Tzb-refractory TzbR cells rechallenged with Tzb. Therefore, the Tzb combination with autophagy inhibitors may be a promising strategy in patients resistant to therapy with Trastuzumab.

### 5.2. Role of Autophagy in the Enhancement of the Inhibitory Effect of Breast Cancer Treatments

#### 5.2.1. Autophagy Enhances the Inhibitory Effect of Paclitaxel through ARHI Expression

ARHI encodes a small GTP-binding protein belonging to the Ras/Rap superfamily, which has the characteristics of a tumor suppressor gene in ovarian and breast cancers, despite sharing 54–59% homology with Ras proto-oncogenes [77], ARHI is expressed in normal breast epithelial cells, but in more than 70% of breast cancers, it is dramatically downregulated. Loss of ARHI expression has been linked to tumor progression from *in situ* to invasive cancer [78]. Paclitaxel, a cytotoxic drug, can inhibit cancer cell growth by inducing apoptosis and G2/M cell-cycle arrest. TSA, an HDAC inhibitor, can activate several tumor suppressor genes and induces autophagy.

Recent evidence showed that ARHI induces autophagy in breast cancer cells. SKBR3 and MDA-MB231 cells, expressing low levels of endogenous ARHI transfected with ARHI, had an increase of LC3 punctate number, which represent the accumulation of LC3 membrane-bound form on autophagic vesicles. Furthermore, it has been observed that TSA treatment enhanced autophagy, but transfection with siRNA-ARHI blocked the effects of TSA, demonstrating that ARHI is essential for autophagy induction [79].

Other results from the same group showed that TSA greatly enhanced the inhibitory effect of paclitaxel and tumors treated with a combination of ARHI and paclitaxel grew significantly more slowly than controls, whereas the individual treatments did not significantly inhibit tumor growth [79].

## 6. Conclusion and Perspectives

We can conclude that autophagy regulation may provide a useful tool to prevent cancer development, limit tumor progression, and increase the efficiency of cancer treatment. This autophagy regulation has to be context dependent, since an autophagy process increased may be necessary to prevent tumor development in individuals at high risk of cancer. But autophagy activity must be reduced when tumor is already established and subjected to the environmental stresses associated with limited angiogenesis, nutrient deprivation, and hypoxia. 

Understanding the signaling pathways involved in autophagy regulation represents a new direction in the development of anticancer therapies. However, the proteins and trafficking mechanisms involved in the autophagosomal maturation step are not completely understood.

The effectiveness of chemotherapeutics is diminished by the fact that they induce toxicity to both normal and cancer cells. Many targeted therapies studies have been conducted to new drugs development with higher therapeutic index. Currently, signaling transduction pathways, tumor angiogenesis, and malignant stem cells are considered prime targets for new therapeutics development.

## Figures and Tables

**Table 1:** Chemotherapeutic agents involved in autophagy induction.

Target	Drugs and reference
Akt	GSK69O693 [37]
Akt	Perifosine [37]
Akt	Triciribine [37]
AMPK	AICAR [38]
AMPK	Metformin [39, 40]
Bcr-Abl	Imatinib [41]
BNIP3	Arsenic trioxide [42]
CamKK	EB1089 (vitamin D) [43]
HDAC	SAHA [39]
mTORC1	Amiodarone [44]
mTORC1	Curcumin [45]
mTORC1	Everolimus [46]
mTORC1	Niclosamide [44]
mTORC1	Perhexiline [44]
mTORC1	Rottlerin [44]
mTORC1	Temsirolimus [47]
VEGF	Sorafenib [48]
